# Pathogenic hyperactivation of mTORC1 by cytoplasmic EP300 in Hutchinson-Gilford progeria syndrome

**DOI:** 10.15698/cst2024.04.295

**Published:** 2024-04-30

**Authors:** Lucille Ferret, Guido Kroemer, Mojgan Djavaheri-Mergny

**Affiliations:** 1 Centre de Recherche des Cordeliers, INSERM UMRS 1138, Sorbonne Université, Université Paris Cité, Équipe labellisée par la Ligue contre le Cancer, Institut universitaire de France, Paris, France.; 2 Metabolomics and Cell Biology Platforms, Institut Gustave Roussy, Villejuif, France.; 3 Faculté de Médecine, Université de Paris Saclay, Paris, France.; 4 Institut du Cancer Paris CARPEM, Department of Biology, Hôpital Européen Georges Pompidou, AP-HP, Paris, France.

**Keywords:** MTORC1, EP300, autophagy, aging

## Abstract

In a recent issue in *Nature Cell Biology*, Sung Min Son *et al.* unveil a novel layer in the regulation of the mTORC1/autophagy axis by EP300 which can undergo nucleocytoplasmic shuttling in response to alterations in nutrient availability. The study highlights that, in Hutchinson-Gilford progeria syndrome, overabundant cytoplasmic EP300 results in mTORC1 hyperactivation and impaired autophagy, potentially contributing to premature and accelerated aging.

Mechanistic target of rapamycin complex 1 (mTORC1) is a multiprotein complex that serves as a pivotal regulator of cell growth, protein synthesis, metabolism and autophagy [1, 2]. This complex compromises mTOR (mechanistic target of rapamycin), RAPTOR (regulatory associated protein of mTOR), GβL (G protein β subunit-like protein)/mLST8 (mammalian lethal with SEC13 protein 8), DEPTOR (DEP-domain-containing mTOR interacting protein) and PRAS40 (40 kDa proline-rich Akt substrate). Overactivation of mTORC1 signaling is frequently associated with key processes associated with aging and age-related diseases [3, 4]. Conversely, life span extension occurs in various model species (such as worms, yeast, flies and mice) during continuous or intermittent inhibition of mTORC1 by genetic or pharmacological manipulations, respectively [5]. Apparently, this antiaging effect of mTORC1 deactivation is mediated through the induction of autophagy [5].

In a recent issue in *Nature Cell Biology*, Sung Min Son *et al.* [6] unveil a novel layer in the regulation of the mTORC1/autophagy axis by the acetyltransferase EP300 that is intricately linked to nucleocytoplasmic shuttling of EP300. The study reveals that Hutchinson-Gilford progeria syndrome (HGPS), a disease associated with accelerated aging, may be explained by enhanced activity of cytoplasmic EP300, resulting in mTORC1 hyperactivation and impaired autophagy.

Several pathways regulate mTORC1 in response to nutrient status [2] (**Fig. 1**). Thus, the abundance of some amino acids (such as leucine and other branched chain amino acids) stimulate mTORC1 activity through EP300-dependent RAPTOR acetylation, a crucial step for the recruitment of cytoplasmic RAPTOR to Rag GTPase on lysosomal membranes [7]. This event facilitates mTORC1 translocation to the surface of lysosomes where it becomes active through its interaction with RHEB. Upon glucose withdrawal, or depletion of other sources of energy, adenosine monophosphate-dependent kinase (AMPK) is activated and represses mTORC1 activity either directly through phosphorylation of RAPTOR or indirectly by phosphorylating Tuberous Sclerosis Complex 2 (TSC2), which is an upstream regulator of mTORC1 [8, 9]. The consequent inhibition of mTORC1 activity then results in decreased phosphorylation of mTORC1 substrates including Unc-51-like autophagy-activating kinase 1 (ULK1), ultimately culminating in the initiation of autophagy [10].

**Figure 1:**
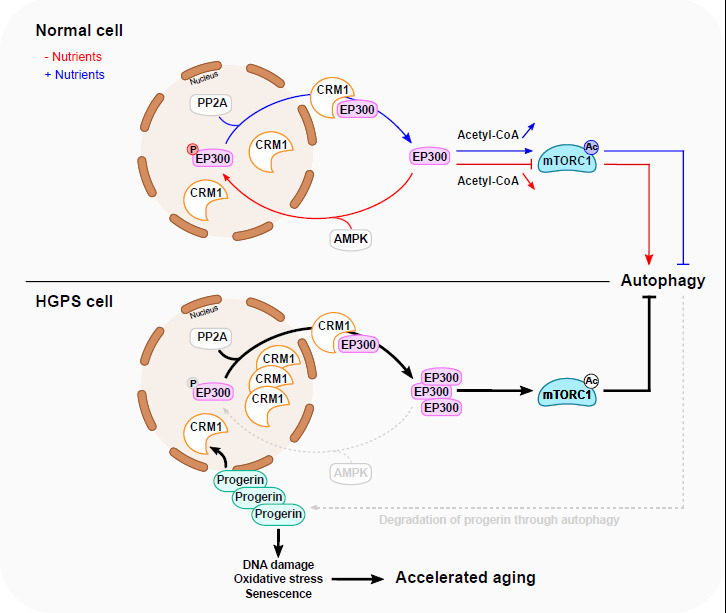
FIGURE 1: A proposed model for EP300 cytoplasm-nucleus shuttling in response to nutrients and in HGPS. Nutrient depletion triggers the relocation of EP300 from the cytoplasm to the nucleus resulting in the inhibition of mTORC1 and subsequent stimulation of autophagy. This is facilitated through AMPK-dependent phosphorylation of EP300 at Serine 89. Nutrients replenishment in starved cells induces PP2A-dependent dephosphorylation of nuclear EP300, facilitating its transportation to cytoplasm by CRM1, thereby promoting mTORC1 reactivation. In HGPS cells, EP300 cytoplasm–nucleus shuttling is altered, causing mTORC1 hyperactivation and impaired autophagy. This is mediated by Progerin-dependent upregulation of CRM1 and alterations in AMPK activity. Interestingly, impaired autophagy leads, in turn, to the accumulation of Progerin, which potentially amplifies the disturbance of the mTORC1/autophagy pathway. The accumulation of Progerin initiates diverse cellular responses linked to accelerated aging. Normalizing HGPS phenotypes can be achieved by modulating EP300 nucleocytoplasmic shuttling. Blue line depicts a nutrient-rich condition, while the red line depicts a nutrient-depleted condition. The grey dotted lines delineate the compromised autophagy process observed in HGPS.

While these findings underscore the critical role of EP300 in regulating mTORC1 activity in response to nutrient status, several questions have remained unanswered: Is EP300-mediated RAPTOR acetylation a rate limiting factor of mTORC1 activation in response to nutrient status? Does nucleocytoplasmic localization of EP300 influence mTORC1 activity? What is the impact of EP300-mediated mTORC1 activation on autophagy? How is this pathway regulated in diseases associated with accelerated aging?

Sung Min Son *et al.* examined these burning questions by exploring the precise mechanism through which EP300 regulates mTORC1 activity after amino acid or glucose deprivation [6]. They showed that EP300 (rather than other lysine acetyltransferases, KATs), plays a critical role in regulating mTORC1 activity through a mechanism that involves the acetylation of the mTORC1 component RAPTOR. In fact, the depletion of EP300 from cells by RNA interference leads to the impairment of mTORC1 activity accompanied by diminished RAPTOR acetylation as well as by decreased lysosomal membrane localization of mTORC1. These deficiencies were effectively rescued by the overexpression of functional wild-type (WT) EP300 but not by a dominant-negative (DN) EP300 construct. Depletion of EP300 also resulted in enhanced functional autophagy, corroborating previous studies underscoring the capacity of EP300 to repress autophagy in response to several stimuli including nutrient starvation, spermidine and curcumin [11, 12]. Conversely, pro-autophagic stimuli either by depletion of cytosolic acetyl coenzyme A (CoA), the donor of acetyl groups transferred onto EP300 substrates, or by direct inhibition of the catalytic activity of EP300, reduced EP300 activity [11, 13].

A large body of evidence supports the involvement of EP300 in the negative regulation of autophagy through the acetylation of various ATG proteins, each playing distinct roles in various steps of the autophagic cascade [14]. However, Sung Min Son *et al.* reported that upon depletion of amino acids, the dominant process resulting in autophagy stimulation involves reduced RAPTOR acetylation leading to mTORC1 inhibition rather than deacetylation of other autophagy regulators [6]. Several questions arise from these findings: How does the localization of EP300 affect the accessibility of EP300 to its diverse substrates? Does EP300-mediated RAPTOR acetylation serve as a universal mechanism for the regulation of autophagy?

More importantly, Sung Min Son *et al.* demonstrated that the cytoplasm-to-nuclear transport of EP300 plays as a central role in regulating mTORC1 in response to nutrient status. The authors showed that EP300 nuclear transport occurs in diverse cell lines exposed to amino acid or glucose deprivation, accompanied by diminished EP300 cytoplasmic activity and reduced interaction between EP300 and RAPTOR [6]. Moreover, the reduction in RAPTOR acetylation and mTORC1 activity in cells expressing a constitutively cytoplasmic form of EP300 construct was less prominent after amino acid or glucose depletion as compared to WT EP300. Similarly, the brains of starved mice, which exhibit decreased RAPTOR acetylation and mTORC1 activity, also contain more nuclear and less cytoplasmic EP300 [6]. Altogether, these findings suggest that the nucleocytoplasmic shuttling of EP300 plays a pivotal role in the regulation mTORC1 activity in response to nutrient status both *in vitro* and *in vivo*.

Moreover, using an acetylation-refractory mutant of RAPTOR in which a critical lysine residue that usually is acetylated by EP300 was replaced by arginine (KR; K1097R). Sung Min Son *et al.* showed that cytoplasmic EP300 represses autophagy through a mechanism that depends on RAPTOR acetylation. The impact of EP300 cellular localization on autophagy regulation has also been reported by Sebti *et al.*, who demonstrated that during starvation, BCL2-associated athanogene 6 (BAT6), binds to EP300 and facilitates its transport to the nucleus [15]. When BAT6 is absent, EP300 nuclear shuttling is inhibited in starved cells, leading to cytoplasmic ATG7 hyperacetylation and subsequently autophagy inhibition. It might be interesting to investigate the precise molecular mechanism through which BAT6 regulates EP300 shuttling and determine its regulation of mTORC1 activity.

Sung Min Son *et al.* investigated how EP300 shuttling is regulated by nutrient status (**Fig. 1**). Among specific inhibitors of major kinases, they showed that only an AMP-activated protein kinase (AMPK) inhibitor could suppress the nuclear transport of EP300 after amino acid depletion. Mechanistically, during amino acid starvation, AMPK phosphorylates EP300 at serine 89, thereby facilitating its nuclear translocation and its trapping by 14-3-3ζ in the nucleus, then leading to decreased RAPTOR acetylation and subsequent inhibition of mTORC1 activity. Conversely, the addition of amino acids to starved cells leads to the dephosphorylation of EP300 by phosphatase 2A (PP2A). This event facilitates the nuclear export of EP300 to the cytoplasm through a mechanism involving the chromosomal maintenance 1 (CRM1), also known as exportin 1.

The Hutchinson-Gilford progeria syndrome (HGPS) is a rare sporadic autosomal dominant disorder characterized by accelerated aging [16]. Earlier studies have demonstrated mTORC1 hyperactivity in HGPS, and inhibition of mTORC1 effectively diminishes characteristic HGPS phenotypes, such as defects in nuclear morphology (with deformation of the envelope) and accumulation of Progerin (the disease-causing protein), suggesting a causal role for mTORC1 overactivation in HGPS [17, 18].

Sung Min Son *et al.* investigated possible alterations of EP300 cytoplasm-nuclear shuttling in HGPS patient-derived fibroblasts. By employing doxycycline-inducible Progerin-expressing cell lines, the authors showed that Progerin expression led to a reduction of EP300 nuclear transport causing mTORC1 activation and subsequently autophagy inhibition. In addition, EP300 nuclear translocation and mTORC1 activity were compromised in fibroblasts derived from HGPS patients after amino acid starvation. Moreover, HGPS fibroblasts failed to activate AMPK following starvation and exhibited increased CRM1 expression [6]. These findings corroborate prior findings indicating an enhanced nuclear protein export pathway in HGPS that is linked to progerin-mediated CRM1 upregulation [19].

Altogether, the results suggest that the overactivation of mTORC1 in HGPS is linked to the alteration of EP300 cytoplasm-nuclear shuttling driven by Progerin (**Fig. 1**). This may be ascribed to CRM1 upregulation and/or alteration in AMPK activity, or potentially involve other yet-to-be-defined mechanisms. Interestingly, the degradation of Progerin through autophagy is compromised in HGPS, suggesting the existence of a vicious cycle in which the elevation of Progerin inhibits autophagy and compromised autophagy then reduces the capacity of cells to keep Progerin levels low. The abnormal accumulation of Progerin in HGPS can be corrected by inhibitors of EP300 or CRM1 or activators of AMPK. Similarly, rapamycin, an inhibitor of mTORC1 and inducer of autophagy, has the capacity to normalize the characteristic HGPS phenotypes [17, 18]. In another study, spermidine, an inhibitor of EP300 that stimulates autophagy, has demonstrated the capability to significantly extend the lifespan of yeast, flies and worms, and human immune cells [12]. Other caloric restriction mimetics (compounds that trigger the protective mechanisms associated with caloric restriction) have also been shown to promote autophagy and reverse aging-derived effects through a reduction of protein acetylation [20].

In conclusion, the work of Sung Min Son *et al.* advances our understanding of how the nucleocytoplasmic shuttling of EP300 affects the mTORC1/autophagy axis in response to nutrient status and in Hutchinson-Gilford progeria syndrome. The authors propose a rationale for targeting EP300 and its regulatory nucleocytoplasmic shuttling mechanisms. Based on this work, direct or indirect EP300 inhibitors may be considered as promising therapeutic targets for the treatment of HGPS. The development of next-generation EP300 inhibitors might also be useful for the prevention or treatment of other progeroid syndromes and age-associated diseases as well as for “normal” aging.
